# Effect of *in vivo* administration of reprogramming factors in the mouse liver

**DOI:** 10.3892/ol.2013.1418

**Published:** 2013-06-20

**Authors:** AKIRA TOMOKUNI, HIDETOSHI EGUCHI, HIROMITSU HOSHINO, DYAH LAKSMI DEWI, SHINPEI NISHIKAWA, YOSHIHIRO KANO, NORIKATSU MIYOSHI, ARINOBU TOJO, SEIICHIRO KOBAYASHI, NORIKO GOTOH, KUNIHIKO HINOHARA, NOEMI FUSAKI, TOSHIYUKI SAITO, HIROSHI SUEMIZU, HIROSHI WADA, SHOGO KOBAYASHI, SHIGERU MARUBASHI, MASAHIRO TANEMURA, YUICHIRO DOKI, MASAKI MORI, HIDESHI ISHII, HIROAKI NAGANO

**Affiliations:** 1 Department of Gastroenterological Surgery, Osaka University Graduate School of Medicine, Suita, Osaka 565-0871;; 2 Divisions of Molecular Therapy, University of Tokyo, Minato-ku, Tokyo 108-8639;; 3 Systems Biomedical Technology, Institute of Medical Science, University of Tokyo, Minato-ku, Tokyo 108-8639;; 4 DNAVEC Corporation, Tsukuba, Ibaraki 300-2611;; 5 Transcriptome Profiling Group, National Institute of Radiological Sciences, Inage-ku, Chiba 263-8555;; 6 Biomedical Research Department, Central Institute for Experimental Animals, Miyamae, Kawasaki, Kanagawa 216-0001, Japan

**Keywords:** *Kras*, *p53*, reprogramming, differentiation, liver

## Abstract

Cancer is initiated by the transformation of stem cells or progenitor cells via a dedifferentiation process that leads to cancer stem cells; however, the process involves the activation of growth-promoting oncogenes and the inactivation of growth-constraining tumor suppressor genes. The introduction of defined factors, such as those encoded by *c-Myc*, *Sox2*, *Oct3*/*4* and *Klf4,* in normal somatic cells results in their dedifferentiation into induced pluripotent stem (iPS) cells. We previously reported that these defined factors induced the development of induced multipotent cancer (iPC) cells from gastrointestinal cancer cells by reducing tumor aggressiveness. Previous studies indicated that although reprogramming may be facilitated by *p53* inhibition, gain-of-function oncogenic mutations in *p53* and oncogenic mutations in *Kras*-stimulated tumorigenic activity, and their roles *in vivo* are imperfectly understood. Hence, in the present study, the effect of direct injection of a Sendai virus (SeV) vector encoding four defined factors *in vivo* was studied using various backgrounds of transgenic and knockout mice, and was compared with that of direct injection of microRNAs (miRNAs) diluted with cationic lipid. The *in vivo* imaging data revealed transformation hot spots for *p53* deficiency or conditional activation of mutant *Kras*, and the sizes were concordant with those in immuno-deficient NOD/SCID and uPA-NOG mice, as well as larger compared with those in the control mice. Overall, the present data on *in vivo* reprogramming indicated that *Kras* activation may facilitate the effect of cellular reprogramming in normal liver cells, and the effect of *Kras* activation is more apparent than that of tumor suppressor *p53* deficiency. The results also revealed that immunodeficiency may increase the effect of reprogramming, presumably by blocking the immunosurveillance of transformed cells. These findings provide a rationale for further studies to develop a therapeutic approach involving direct *in vivo* reprogramming.

## Introduction

The discovery that complete cellular reprogramming may be achieved by introducing the defined transcription factors *c-Myc*, *Sox2*, *Oct3*/*4* and *Klf4* into terminally differentiated somatic fibroblasts of mouse and human origins was an important breakthrough (1,2). The generation of induced pluripotent stem (iPS) cells by the introduction of defined factors, which are generally expressed in embryonic stem (ES) cells, results in the reconstitution of organs in chimeric mice and contributes to the regeneration of human tissues (3). We previously showed that gastrointestinal cancer cells acquired multipotential differentiation ability upon the introduction of defined factors; the gene expression profiles of mesodermal and ectodermal cells appeared in gastrointestinal cancer cells of endodermal origin [termed induced multipotent cancer (iPC) cells] (4). Whether the iPC cells were generated via a state of pluripotency remains to be investigated, although the iPC cells expressed ES-like genes and possessed the ability to differentiate from cells of endodermal origin into other endoderm and mesoderm lineages (4). Notably, *in vitro* differentiation resulted in sensitization to therapeutic reagents such as vitamins A and D and the chemotherapeutic agent 5-fluorouracil (5-FU), as well as reduced tumorigenicity, suggesting that altering the cancer cell lineage through reprogramming *in vivo* may be a promising concept for novel and efficient cancer therapy (4). However, at present, there are a limited number of studies concerned with reprogramming *in vivo,* and thus the mechanism involved in reprogramming *in vivo* remains unknown.

Epithelial tumor tissues are composed of various types of mesenchymal cells, such as myofibroblasts, fibroblasts, endothelial cells, lymphocytes, monocytes and macrophages, certain of which are known to be components of a microenvironment (niche). These components are involved in tumorigenesis at the early stages, support cancers cells and provide resistance against exposure to chemotherapeutic reagents. Overall, although it is assumed that mesenchymal cells are important in the process of reprogramming in the complex system *in vivo*, no investigations on how reprogramming factors affect the mesenchymal components have been conducted. To assess this, the effect of direct injection of a Sendai virus (SeV) vector encoding four defined factors into the liver was studied using transgenic and knockout mice with various genetic backgrounds, and the effect was compared with that of direct injection of microRNAs (miRNAs) diluted with cationic lipid. The *in vivo* bioluminescence imaging data revealed transformation hot spots for *p53* (also known as *TP53* in humans and *Trp53* in mice) deficiency or conditional activation of mutant *Kras,* and the sizes were consistent with those in immunodeficient NOD.CB17-*Prkdc^scid^*/J (NOD/SCID) mice and NOD.Cg-*Prkdc^scid^Il2rg^tmSug^*/Jic (NOG) mice expressing transgenic urokinase-type plasminogen activator (*uPA*) in the liver (uPA-NOG), as well as larger compared with those in the control mice. The present results suggested that the effect of reprogramming-based, novel therapeutic approaches was enhanced by *Kras* activation. The effect was more apparent with *Kras* activation than with tumor suppressor *p53* deficiency, suggesting a distinct role for the *Kras* pathway in direct reprogramming in the liver. Furthermore, immunodeficiency may increase the effect of reprogramming, presumably by blocking the immunosurveillance of transformed cells.

## Subjects and methods

### Experimental animals

NOD/SCID mice were purchased from Charles River Japan (Osaka, Japan). All animal experiments were performed with approval from the Animal Experiments Committee of Osaka University. The NOD/SCID mice lack B cells, T cells and the complement system, and possess severely reduced natural killer (NK) cells. More severely immunodeficient uPA-NOG mice were produced by extra-uterine fertilization, resulting in zygotes that expressed transgenic *uPA* in the liver; the extracellular matrix in the liver was modified to activate the hemolytic system, which facilitated xenogeneic engraftment or growth of transformed cells in the present experiment in mice with an immunodeficient background (5). Heterozygous B6.129S4-*Kras^tm4Tyj^*/J mice (Jackson Laboratory, Bar Harbor, ME, USA), which carry an allele with the most common point mutation whose expression is blocked by the presence of a loxP-flanked stop codon in the ROSA loci, were crossed with B6129-Tg(MMTV-Cre)4Mam/J mice (Jackson Laboratory), which express P1 Cre recombinase under the control of the mouse mammary tumor virus (MMTV) long terminal repeat (LTR) promoter. The MMTV LTR promoter directs a widespread pattern of expression to produce CMV-Cre/*Kras^mut^* mice; and when expressed in B6.Cg-Tg(Alb-Cre)21Mgn/J mice (Jackson Laboratory), is efficient in achieving liver-specific recombination to produce Alb-Cre/*Kras^mut^* mice. B6.129S2-*Trp53^tm1Tyj^*/J mice (Jackson Laboratory), from which a mutant allele was produced by a targeted neo insertion into the *p53* locus, were mated with STOCK Tg(Nanog-GFP, Puro)1 Yam mice, which express the green fluorescent protein under the control of the *Nanog* gene promoter (RIKEN BioResource Center, Tsukuba, Japan), to produce Nanog-GFP/*Trp53^+^*^/^*^− (KO)^* mice. Overall, two immunodeficient mice were used in the experiments, NOD/SCID and uPA-NOG, as well as CMV-Cre/*Kras^mut^*, Alb-Cre/*Kras^mut^* and Nonog-GFP/*Trp53^KO^* mice. miRNAs were also used to assess the effect.

### In vivo administration of viral construct mixture

SeV vectors replicate in the form of negative-sense single-stranded RNA in the cytoplasm of infected cells and do not undergo a DNA phase or integrate into the host genome (6). It was shown that the efficient induction of transgene-free human pluripotent stem cells was achieved using a vector based on SeV, an RNA virus that does not integrate into the host genome; iPS induction could be achieved by the SeV-mediated gene-transfer introduction of the defined transcription factors *c-Myc*, *Sox2*, *Oct3*/*4* and *Klf4* from terminally differentiated somatic cells (7). A viral construct mixture consisting of: i) 5 *μ*l lentiviral vector and ii) SeV vectors (2.5 *μ*l per each transcription factor) or 10 *μ*l miRNAs was prepared. Co-transfection of the lentiviral luciferase gene was performed to trace the cell populations in which the genes were introduced. The SeV vectors were mixed according to the transcription factors to be introduced, such as SeV vectors encoding *c-Myc*, *Sox2*, *Oct3*/*4* and *Klf4* (MSOK); *Sox2*, Oct4 and *Klf4* (SOK); or *c-Myc* alone (M). With regard to miRNAs, 60 pmol of double-stranded mature miRNAs (20 pmol of mmu-miR-200c; 5 pmol of mmu-miR-302a, -302b, -302c and -302d; and 10 pmol of mmu-miR-369-3p and -5p) was diluted with 10 *μ*l siPORT (Ambion, Austin, TX, USA). Median laparotomy was performed in each mouse under sevoflurane anesthesia and the viral construct mixture was directly injected into the median lobe of the liver.

### In vivo imaging

**Figure 1.**
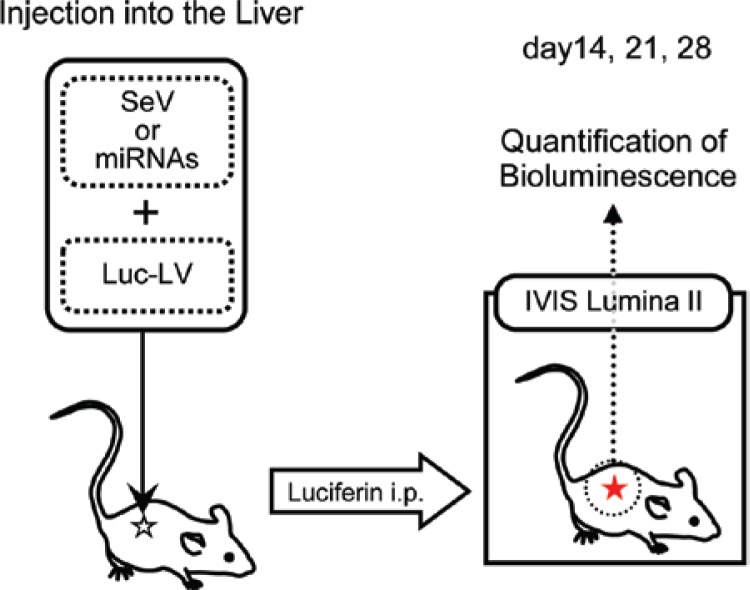
Schematic representation of the study. The immunodeficient mice, NOD/SCID and uPA-NOG, as well as the CMV-Cre/*Kras^mut^*, Alb-Cre/*Kras^mut^* and Nonog-GFP/*Trp53^KO^* mice, received injection of the Sendai virus (SeV) vector encoding transcription factors (including *c-Myc*, *Sox2*, *Oct3/4* and *Klf4)* into the livers by laparotomy. As a control, microRNAs (miRNAs) diluted with cationic lipid were injected. To trace the effect, the lentiviral luciferase gene (Luc-LV) was co-injected. At the indicated days after injection, the mice received administration of luciferin intraperitoneally and the signal was assessed using the IVIS Lumina II system.

To trace the behavior of the injected viral construct, the animals were examined at days 14, 21 and 28 using the IVIS Lumina II imaging system (Caliper Life Sciences, Hopkinton, MA, USA) (Fig. 1). Each mouse received luciferin intraperitoneally at 4 mg/kg and was then anesthetized with 2% isoflurane; the mice were left undisturbed for 10 min thereafter. Subsequently, the mice were imaged under the following conditions: Exposure, 2 min; f-stop, 1; binning, medium; field of view, 12.5 cm. Bioluminescence values were calculated as photons/s/cm^2^/sr in the region of interest.

## Results

### Immunodeficient mice

**Figure 2.**
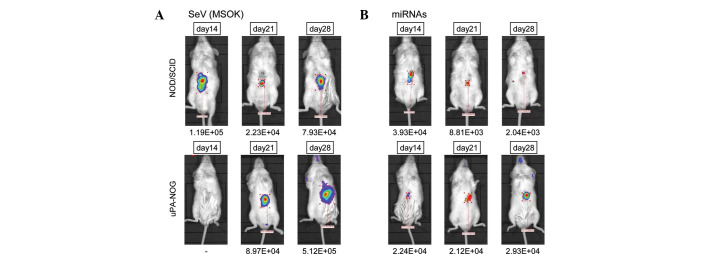
Immunodeficient mice with reprogramming factors. Two immunodeficient mice, NOD/SCID and uPA-NOG, received four factors (*c-Myc, Sox2, Oct3/4 and Klf4*; MSOK) or microRNAs (miRNAs). At the indicated days after injection, the mice received luciferin and the signal was assessed using the IVIS Lumina II system. The colored area represents the luciferase-positive area and its bioluminescence was quantified as shown below the respective images. SeV, Sendai virus.

In the NOD/SCID mice, the luciferase-positive area was detected 14, 21 and 28 days following the injection of viral construct mixture (Fig. 2A). The mice showed no apparent health problems. The uPA-NOG mice showed a more apparent luciferase-positive area, which was negative at day 14, but positive at day 21 and more apparent compared with day 28. The data suggested that liver-specific modification of the extracellular matrix under immunodeficient conditions may induce a more apparent effect. By contrast, direct injection of miRNAs indicated that the luciferase-positive area was relatively small in NOD/SCID mice, but was increased in uPA-NOG mice at day 28 (Fig. 2B), suggesting that the effects of the SeV vector infection were more apparent than the *in vivo* transfection of miRNAs, and that the extracellular structure of the liver and immunosurveillance may alter the effect.

### Oncogenic Kras activation in mice

**Figure 3.**
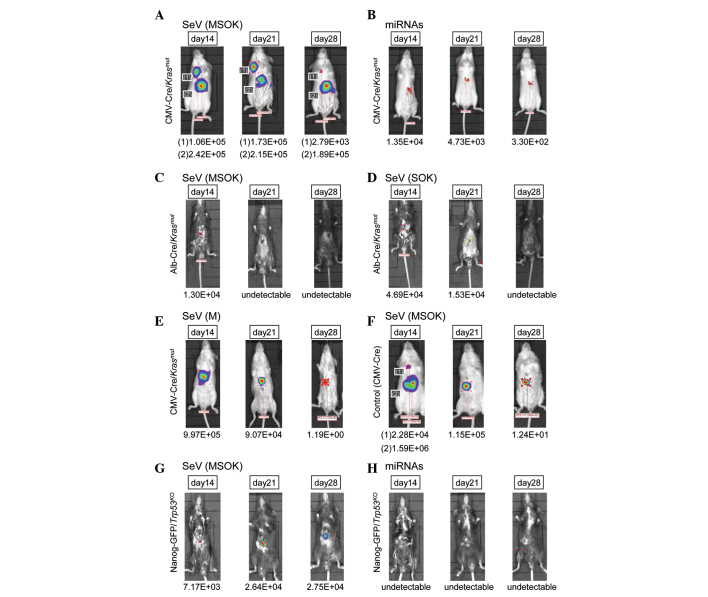
Oncogenic *Kras*-expressing mice and tumor suppressor *p53*-deficient mice with reprogramming factor(s) or microRNAs (miRNAs). (A–F) Two conditional knockout mice, CMV-Cre/*Kras^mut^* and Alb-Cre/*Kras^mut^* and the control CMV-Cre mice received four factors (*c-Myc, Sox2, Oct3/4 and Klf4*; MSOK), three factors (*Sox2*, *Oct3/4* and *Klf4*; SOK), one factor (*c-Myc*; M) or miRNAs. Tumor suppressor *p53*-deficient mice received (G) four factors (MSOK), or (H) miRNAs. At the indicated days after injection, the mice received luciferin and the signal was assessed using the IVIS Lumina II system. The colored area represents the luciferase-positive area and its bioluminescence was quantified as shown below the respective images. SeV, Sendai virus.

To investigate the effect of oncogenic *Kras* activation in mice, CMV-Cre/*Kras^mut^* mice were produced, which expressed the oncogenic *Kras* allele with a point mutation (G12D; Fig. 3A). The luciferase-positive area was detected at days 14, 21 and 28. Another luciferase-positive area, in the right thoracic region, was also noted. The data suggested that oncogenic *Kras* may be involved in accelerating the cellular reprogramming process. The effect was marginal in miRNA-injected mice (Fig. 3B), presumably due to the relatively low gene transfection efficiency compared with SeV vector injection.

To clarify whether hepatocytes or non-hepatocytes (such as mesenchymal cells) in the liver were involved in the effect, Alb-Cre/*Kras^mut^* mice were produced and SeV vector encoding *c-Myc*, *Sox2*, *Oct3/4* and *Klf4* (MSOK) was directly injected (Fig. 3C). The luciferase-positive area was limited compared with that of CMV-Cre/*Kras^mut^* mice. The data were similar following the injection of SeV vector encoding *Sox2*, *Oct3*/*4* and *Klf4* but not *c-Myc* (SOK; Fig. 3D), suggesting that Alb-positive hepatocytes were unlikely to be targets of cellular reprogramming.

To study the effect of *c-Myc* in oncogenic *Kras* mutation, SeV vector encoding *c-Myc* (M) was injected into CMV-Cre/*Kras^mut^* mice (Fig. 3E). The luciferase-positive area was detected at days 14, 21 and 28, while the injection of SeV vector encoding *c-Myc*, *Sox2*, *Oct3*/*4* and *Klf4* (MSOK) into the control CMV-Cre mice showed a similar luciferase-positive area (Fig. 3F). The data suggested that the oncogenic *Kras* mutation was compatible with the administration of *Sox2*, *Oct3*/*4* and *Klf4*.

### Tumor suppressor p53-deficient mice

Previous studies have shown that the inhibition or absence of *p53* significantly increased the reprogramming efficiency of somatic cells to reach a pluripotent state (8–10). Further studies have demonstrated that decreasing the level of the tumor suppressor *p53* protein enables the development of iPS cells from murine fibroblasts; these iPS cells are capable of generating germ-line-transmitting chimeric mice, suggesting that *p53* may not be necessary for reprogramming. The inhibition or absence of *p53* significantly increases the reprogramming efficiency of human somatic cells (8–10).

To assess the effect of this observation *in vivo*, Nanog-GFP/*Trp53^KO^* mice were produced and infected with SeV vector encoding *c-Myc*, *Sox2*, *Oct3*/*4* and *Klf4* (Fig. 3G). Although the efficiency was low, it was possible to detect the luciferase-positive area at days 14, 21 and 28. The administration of miRNAs did not produce a luciferase-positive area, suggesting that the efficiency of this approach was low or undetectable (Fig. 3H). The data showed that although the effect of *p53* was significant in cellular reprogramming, its effect in direct reprogramming in the liver was limited.

## Discussion

Although there is little knowledge concerning the mechanism of reprogramming *in vivo*, it is known that certain types of gene alterations have significant effects on cellular reprogramming *in vitro*. For example, the absence of *p53*, which is critical in epithelial tumors, increases the efficiency of iPS cell generation (8–10). We previously demonstrated that the reprogramming efficiency was enhanced by co-transfection of key tumor suppressor gene mutants (11, data not shown). The results support the theory that mutations involved in DNA contact may be critical in the efficiency of iPS generation, and suggest two roles for *p53* mutations in reprogramming. Structural mutations may contribute to the maintenance of genomic stability, while DNA contact mutations define the downstream target genes, which may be distinct from wild-type *p53* function. Moreover, in a further reprogramming study using other cancer cells with gain-of-function mutations, such as *p53*R175H and *Kras^G12D^*, we demonstrated the multipotency of differentiation and temporal suppression of tumorigenicity. However, the cells subsequently resumed growth in long-term culture (>2 months) and also showed increased tumorigenicity. After iPS factor-mediated reprogramming, the expression of ES-like genes, with the exception of activated endogenous *c-Myc*, was downregulated in long-term cultures of iPC cells derived from cholangiocellular carcinoma HuCC-T1 cells with gain-of-function mutations. This suggests a role for such oncogenic mutations in the reactivation of a malignant phenotype in long-term culture, presumably via the accumulation of further mutations or increased genomic instability during *in vitro* culture (11).

The present study showed that the following factors were involved in the efficiency of the causal effects due to directly administered reprogramming factors in the liver *in vivo*: i) immunodeficiency; ii) extracellular components such as *uPA*; and iii) activation of oncogenic *Kras* in mesenchymal cells.

Severely immunodeficient NOG mice are utilized as recipients for human tissue transplantation, which produces chimeric mice with various types of human tissue. In the present study, uPA-NOG mice were used. Human hepatocytes injected into uPA-NOG mice repopulated the recipient livers with human cells, and the uPA-NOG model has a number of advantages over previously produced chimeric mouse models of the human liver (5). The immunodeficient condition facilitates this process by the elimination of transformed cells. In the present study, uPA-NOG mice showed larger luciferase-positive areas in comparison with NOD/SCID mice, suggesting that the extracellular matrix has a critical effect on reprogramming. Furthermore, the tissues were examined and an irregular arrangement of hepatocytes was observed, although no cancerous cells or teratoma were detected, suggesting that the cells directly affected by reprogramming factors *in vivo* may be altered or adapted in tissues with a supportive surrounding microenvironment.

Oncogenic *Kras* has a pivotal role in the carcinogenesis and progression of gastrointestinal tumors, such as those of the pancreas and colon, and in novel treatment options in *Kras*-mutant metastatic colorectal cancer. However, *Kras* mutations associated with vinyl chloride exposure and the observed mutations in liver cancers are relatively rare in direct DNA-sequencing analyses following microdissection, suggesting that activation of the oncogenic *Kras* is unlikely to have a significant role in liver cancer (12–15). This is in agreement with the present observation that Alb-Cre/*Kras^mut^* mice, in which the oncogenic *Kras* is activated in Alb-positive hepatocytes, developed a weak luciferase signal. The present data showed a low frequency of luciferase-positive cells in Alb-Cre/*Kras^mut^* mice compared with CMV-Cre/*Kras^mut^* mice, suggesting that Alb-negative cells may be targets of *in vivo* reprogramming. Activating mutations in the *Kras* gene are commonly detected in certain, but not all, types of epithelial cancer. Ray *et al* studied a Cre-mediated *Kras^G12D^* mutation, which has the same position of amino acid substitution as in the present study, during recombination in tissues expressing cytokeratin 19 to understand the susceptibility of various epithelial tissues to *Kras*-induced tumorigenesis (16). The study showed that exposure to extracellular components promoted *Kras^G12D^*-initiated tumorigenesis, although environmental exposure did not consistently correlate with tumor formation, such as that in the small intestine, suggesting the presence of intrinsic differences in susceptibility to *Kras* activation and that tumor susceptibility is not limited to the epithelial cells but is different depending on the cellular context (16). To the best of our knowledge, the present study is the first to demonstrate that the effect of reprogramming factors *in vivo* is not dominant in epithelial cells; instead, the effect is more likely to be transformed in non-epithelial, mesenchymal cells, demonstrating that the efficiency at the same dose is dependent on the cell of origin. However, tumor suppressor *p53* deficiency had limited significance in the present study. Given that the data indicated Alb-negative cell involvement in direct reprogramming in the liver in the present system, genomic surveillance of *p53* may be limited in mesenchymal cells. It is reasonable to consider that the genotype of the *p53*-deficient mice was heterogeneous for *p53* (*p53^+^*^/^*^−^*); thus, the remaining intact allele may be involved in the suppression of the transformation in mice with this genetic background.

The present data indicated that the activation of oncogenic signals, such as *Kras^G12D^*, in mesenchymal tissues may be critical in the generation of the effect of directly administered reprogramming factors in the liver *in vivo*. This may provide answers to queries regarding reprogramming, including efficiency and tumorigenicity, to establish experimental models of organ/tissue/cell-specific oncogenic gain-of-function with various types of immunodeficient mice. Therefore, in the future, a reprogramming-based, novel therapeutic approach may be applied clinically.

## Abbreviations:

iPS cells: induced pluripotent stem cells;
ES cells: embryonic stem cells;
iPC cells: induced multipotent cancer cells;
5-FU: 5-fluorouracil;
SeV: Sendai virus;
miRNA: microRNA;
NOD/SCID mice: NOD.CB17-*Prkdc*^scid^/J mice;
NOG mice: NOD.Cg-*Prkdc**^scid^**Il2rg**^tmSug^*/Jic mice;
uPA: urokinase-type plasminogen activator;
MMTV: mouse mammary tumor virus;
LTR: long terminal repeat

## References

[1] Takahashi K, Yamanaka S (2006). Induction of pluripotent stem cells from mouse embryonic and adult fibroblast cultures by defined factors. Cell.

[2] Takahashi K, Tanabe K, Ohnuki M, Narita M, Ichisaka T, Tomoda K, Yamanaka S (2007). Induction of pluripotent stem cells from adult human fibroblasts by defined factors. Cell.

[3] Yamanaka S (2009). Elite and stochastic models for induced pluripotent stem cell generation. Nature.

[4] Miyoshi N, Ishii H, Nagai K (2010). Defined factors induce reprogramming of gastrointestinal cancer cells. Proc Natl Acad Sci USA.

[5] Suemizu H, Hasegawa M, Kawai K (2008). Establishment of a humanized model of liver using NOD/Shi-scid IL2Rgnull mice. Biochem Biophys Res Commun.

[6] Lamb RA, Kolakofsky D, Knipe DM, Howley PM (2001). Paramyxoviridae; the viruses and their replication. Fields Virology.

[7] Fusaki N, Ban H, Nishiyama A, Saeki K, Hasegawa M (2009). Efficient induction of transgene-free human pluripotent stem cells using a vector based on Sendai virus, an RNA virus that does not integrate into the host genome. Proc Jpn Acad Ser B Phys Biol Sci.

[8] Zhao Y, Yin X, Qin H (2008). Two supporting factors greatly improve the efficiency of human iPSC generation. Cell Stem Cell.

[9] Kawamura T, Suzuki J, Wang YV (2009). Linking the p53 tumour suppressor pathway to somatic cell reprogramming. Nature.

[10] Hong H, Takahashi K, Ichisaka T (2009). Suppression of induced pluripotent stem cell generation by the p53–p21 pathway. Nature.

[11] Nagai K, Ishii H, Miyoshi N (2010). Long-term culture following ES-like gene-induced reprogramming elicits an aggressive phenotype in mutated cholangiocellular carcinoma cells. Biochem Biophys Res Commun.

[12] Feldmann G, Beaty R, Hruban RH, Maitra A (2007). Molecular genetics of pancreatic intraepithelial neoplasia. J Hepatobiliary Pancreat Surg.

[13] Prenen H, Tejpar S, Van Cutsem E (2010). New strategies for treatment of KRAS mutant metastatic colorectal cancer. Clin Cancer Res.

[14] Laurent-Puig P, Zucman Rossi J (2006). Genetics of hepatocellular tumors. Oncogene.

[15] Tannapfel A, Sommerer F, Benicke M (2003). Mutations of the BRAF gene in cholangiocarcinoma but not in hepatocellular carcinoma. Gut.

[16] Ray KC, Bell KM, Yan J, Gu G, Chung CH, Washington MK, Means AL (2011). Epithelial tissues have varying degrees of susceptibility to Kras(G12D)-initiated tumorigenesis in a mouse model. PLoS One.

